# Exploring Calcium Alginate-Based Gels for Encapsulation of *Lacticaseibacillus paracasei* to Enhance Stability in Functional Breadmaking

**DOI:** 10.3390/gels10100641

**Published:** 2024-10-08

**Authors:** Daiva Zadeike, Zydrune Gaizauskaite, Loreta Basinskiene, Renata Zvirdauskiene, Dalia Cizeikiene

**Affiliations:** Department of Food Science and Technolgy, Faculty of Chemical Technology, Kaunas University of Technology, 50254 Kaunas, Lithuania; zydrune.gaizauskaite@ktu.lt (Z.G.); loreta.basinskiene@ktu.lt (L.B.); renata.zvirdauskiene@ktu.lt (R.Z.); dalia.cizeikiene@ktu.lt (D.C.)

**Keywords:** microencapsulation, alginate gel matrix, chitosan coating, bacterial survivability, breadmaking

## Abstract

This study focuses on evaluating the efficiency of acid-tolerant *Lacticaseibacillus paracasei* bacteria encapsulated in an alginate-based gel matrix during repeated sourdough fermentation cycles, as well as their preservation during storage and throughout baking at high temperature. A double-coating procedure was applied, involving the encapsulation of bacterial cells in calcium alginate, which was further coated with chitosan. The encapsulation efficiency (EE) did not show significant difference between alginate and alginate–chitosan (97.97 and 96.71%, respectively). The higher number of *L. paracasei* bacteria was preserved in double-coated microbeads, with survivability rates of 89.51% and 96.90% in wet and dried microbeads, respectively. Encapsulated bacteria demonstrated effective fermentation ability, while double gel-coated cells exhibited slower acidification during sourdough fermentation, maintaining higher efficiency in the second fermentation cycle. The addition of freeze-dried, alginate-based gel-encapsulated bacteria (2–4%, *w*/*w* flour) significantly (*p* < 0.05) improved bread quality and extended its shelf life. A double-layer coating (alginate–chitosan) can be introduced as an innovative strategy for regulating the release of lactic acid bacteria and optimizing fermentation processes. Powdered alginate or alginate–chitosan gel-based *L. paracasei* microcapsules, at appropriate concentrations, can be used in the production of baked goods with acceptable quality and sensory properties, achieving a lactic acid bacteria count of approximately 10^6^ CFU/g in the crumb, thereby meeting the standard criteria for probiotic bakery products.

## 1. Introduction

The global probiotics market is driven by multiple factors, including increased consumer awareness of the health benefits associated with probiotics [1]. Probiotics are now being incorporated into a diverse array of foods and beverages beyond traditional dairy products [2]. Bread remains a staple food in many cultures, constituting a significant portion of the average diet by supplying essential energy and nutrients. Nonetheless, the development of functional baked goods containing probiotics has encountered challenges, primarily due to the high temperatures involved in baking.

Maintaining cell viability during baking and storage is crucial, as probiotic foods must contain approximately 10^6^–10^7^ CFU/g of live bacteria at the time of consumption to exert health benefits [3]. Nevertheless, there are challenges associated with the use of probiotics in baked goods, such as bacterial strain growth, stability in the gastrointestinal environment, processing temperatures, and storage conditions [4]. To enhance the survival of probiotic cells under adverse processing and storage conditions, microencapsulation in hydrogel beads is typically employed [5,6].

The methodologies, as well as the coating materials used for microencapsulation, have to preserve the vitality of microorganisms during the encapsulation process [6,7]. Among the natural raw materials used to obtain microparticles, alginates are the most popular and deserve special attention. In the food industry, alginate-based gel formulations are used as texture modifiers or to improve the stability and long-term efficacy of bioactive compounds [8,9].

Numerous studies in recent years have explored alginate-based materials for the encapsulation of various lactic acid bacteria (LAB) and enzymes. Alginate-based gels have been used for enzyme encapsulation to improve stability and to analyze release mechanisms [10]. The encapsulation of LAB primarily focuses on enhancing bacterial viability and their resistance to acidic gastrointestinal conditions, often employing co-encapsulation with other materials [11]. Encapsulation within an alginate-based gel matrix has been shown to provide better survival rates compared to free cells during gastric transit and other harsh environmental conditions. For example, Trabelsi et al. demonstrated the use of alginate and alginate–chitosan coatings to improve the survival of probiotic bacteria in gastric juice [12], while Mahmoud and colleagues explored the survivability of alginate-microencapsulated *Lactiplantibacillus plantarum* during storage and simulated food processing conditions [13]. However, while these studies provide valuable insights into bacterial survival under gastrointestinal or storage conditions, they do not fully address the challenges of applying encapsulated bacteria in high-temperature environments like baking.

To our knowledge, there are few studies on the use of encapsulated LAB in the production of sourdough and probiotic bread. Ghasemi et al. investigated the impact of encapsulated *L. plantarum* and *L. acidophilus* in tragacanth gum and sago starch on the physicochemical properties of gluten-free sorghum bread and the viability of these bacteria after baking and during staling [14]. In another study, Denkova and co-authors used probiotic strains of lactobacilli for the development of sourdough starters immobilized and freeze-dried using a combined hydrocolloid matrix—high-ester pectin and sodium alginate—for wheat and rye bread [15]. Zhang et al. [16] examined the effects of baking conditions and storage on the viability of *L. plantarum* in bread. Caglar, Ermis, and Durak investigated drying techniques, such as spray-drying and freeze-drying, to provide longer shelf-life of bread sourdough [17]. The mentioned drying methods could be employed to produce stabilized sourdough powders [18]. However, there is a lack of information in the literature related to freeze-dried sourdough and encapsulated bacteria, including evaluation of their use in breadmaking.

This study aims to evaluate the potential of acid-tolerant *Lacticaseibacillus paracasei* bacteria encapsulated in alginate-based gel microcapsules for fortifying wheat baked goods with probiotics. The research focuses on improving the efficiency of LAB across multiple applications, including repeated sourdough fermentation cycles, the stability of both wet and freeze-dried microcapsules during storage, and maintaining probiotic viability throughout high-temperature baking processes, thereby addressing existing encapsulation strategies.

## 2. Results and Discussion

### 2.1. The Encapsulation Efficiency and Morphology of the Alginate-Based Gel Microcapsules

**Figure 1:**
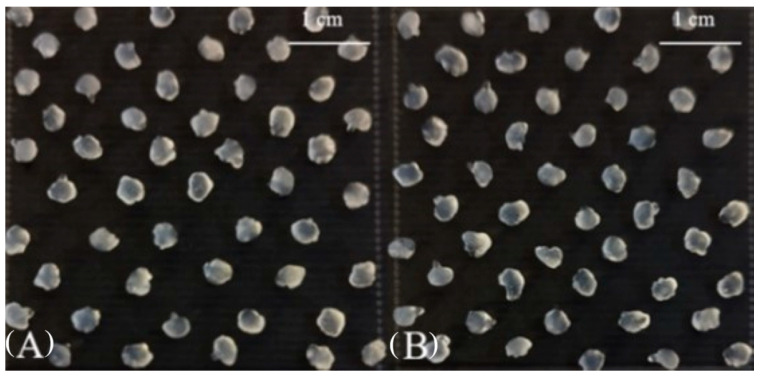
The photo images of alginate gel- (**A**) and alginate–chitosan-coated (**B**) microcapsules.

The encapsulation efficiency (EE) was evaluated based on the logarithmic value of the bacterial count in alginate-gel-based microcapsules, compared to the initial logarithmic value of the cell count before encapsulation (Table 1), as well as the shape and size of the microcapsules (Figure 1).

#### 2.1.1. Encapsulation Efficiency

Encapsulation efficiency (EE) is one of the most important parameters for determining the efficacy of the encapsulation process and the selected encapsulating agent. Our study demonstrates that EE of the alginate and alginate–chitosan capsules loaded with *L. paracasei* ranged between 97% and 98% (the initial logarithmic count of bacterial cells in the encapsulation solution was 10.35 log_10_ CFU/mL) (Table 1). Although 18.5% more alginate–chitosan microcapsules by weight were obtained after the encapsulation procedure compared to those of alginate, otherwise, there was not a significant difference (*p* ≥ 0.05) between the EE values using alginate and alginate–chitosan (Table 1).

The high EE values (>90%) can be explained by the fact that a cell culture taken from the stationary phase exhibits better survivability than that from the log phase [19]. The mild encapsulation operation conditions could also explain the effective encapsulation [13].

The results imply that the coating methods had no effect on *L. paracasei* cell viability and are in agreement with the results reported by other authors. In the study of Oberoi et al. [7], microbeads loaded with *Lacticaseibacillus rhamnosus* had an encapsulation efficiency ranging between 70.06 and 95.13%, which depended on the encapsulation material used. The authors reported that the alginate and xanthan gum composition exhibited the best EE (95.13%), alongside alginate–starch and alginate–gum acacia (93.8 and 93.06%) microbeads. In the study by Zubair et al., the EE of *L. rhamnosus* in calcium alginate was 81.78% [20]. On the other hand, the lowest *L. rhamnosus* encapsulation efficiency of 70.06% was obtained using alginate–chitosan microencapsulation systems via extrusion [7].

In another case, Krasaekoopt and co-authors reported high cell loading in the range of 9.0–9.2 log_10_ CFU/g microbeads in uncoated alginate and chitosan-coated alginate beads, representing encapsulation efficiency of 91.9–99.9% [21]. In our study, both alginate and alginate–chitosan microencapsulation systems also indicated a promising approach for loading LAB cells.

#### 2.1.2. The Shape and Size of Wet Alginate Gel Microcapsules

In the case of morphological analysis, the obtained alginate and alginate–chitosan microcapsules were of oval or spherical shape with multiple cavities and a wrinkled surface (Figure 1). In both cases, there were significant (*p* < 0.05) differences between the size of the beads: the size of the alginate and alginate–chitosan capsules ranged between 2.0 and 3.8 mm, and between 3.0 and 4.4 mm, respectively (see Table 1).

The obtained results align with findings from other studies, which indicate that the encapsulation matrix and method significantly influence the size of microbeads [22]. According to the literature, encapsulation of probiotics using the extrusion method can produce beads with sizes ranging between 0.5 and 3 mm [23]. In a study by Gandomi et al. [24], the average size of alginate–chitosan microbeads, both with and without inulin, was 1.40 and 1.39 mm, respectively. Lotfipour et al. [25] produced microcapsules containing *Lacticaseibacillus acidophilus* DMSZ20079 with sizes ranging from 1.59 to 1.67 mm for alginate and from 1.61 to 1.80 mm for alginate–psyllium, achieving an encapsulation efficiency of over 98%. Another study by Parsana et al. [26] demonstrated that the diameter of various microcapsules containing *Limosilactobacillus reuteri* SW23 or *Ligilactobacillus salivarius* RBL50 cells ranged from 2.1 to 2.8 mm.

The size of microcapsules is of major importance in probiotic bacteria protection. Heidebach et al. [27] showed that a range of particle sizes between 0.2 mm and 3 mm can provide strong protection for probiotics during gastrointestinal exposure. Krasaekoopt and Watcharapoka noted that coating alginate microbeads with chitosan increased the bead size by up to 17%, attributed to the formation of a polyelectrolyte membrane on the surface of the alginate capsule due to electrostatic interactions between alginate and chitosan, resulting in slightly larger coated capsules compared to uncoated ones, which can be translated into increased probiotic protection [28]. The coating has the ability to reduce the permeability of the capsule, and implicitly the oxygen exposure of the probiotics, therefore increasing their stability under harsh conditions, such as high temperatures and low pH [29].

#### 2.1.3. Microstructure and Size Distribution of Freeze-Dried Alginate-Based Gel Capsules

Looking for a solution for more efficient preservation of encapsulated bacterial cells, some of the microcapsules were freeze-dried. The stabilization procedure reduced the count of *L. paracasei* cells by 0.64 and 0.82 log units in the freeze-dried alginate and alginate–chitosan microbeads, respectively (Table 1).

**Figure 2:**
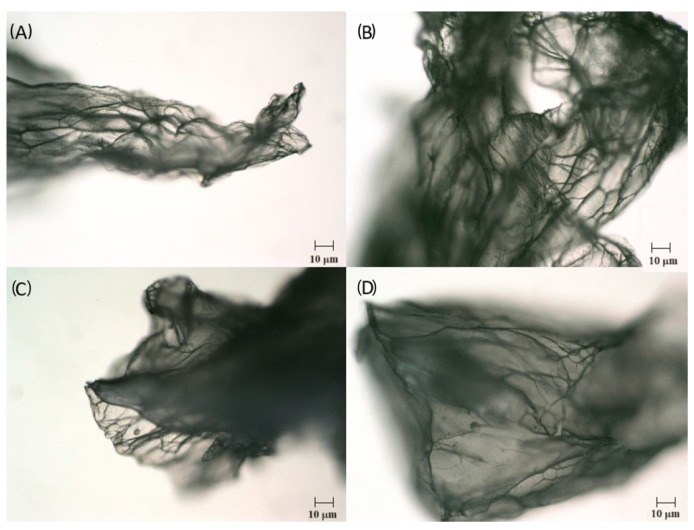
The freeze-dried alginate–chitosan (**A**,**B**) and alginate (**C**,**D**) microcapsules (optical microscope, 100×).

Optical microscopy showed that the freeze-dried microcapsules exhibited surface irregularities as did the wet capsules, but there were no significant differences noticed between the microstructure of alginate and alginate–chitosan microbeads (Figure 2).

According to the literature [30], calcium alginate swells greatly in an aqueous solution, losing its ability to maintain a spherical structure due to dehydration during lyophilization. It was also reported that chitosan-coated capsules retained a more spherical shape after freeze-drying compared to uncoated beads [31].

**Figure 3:**
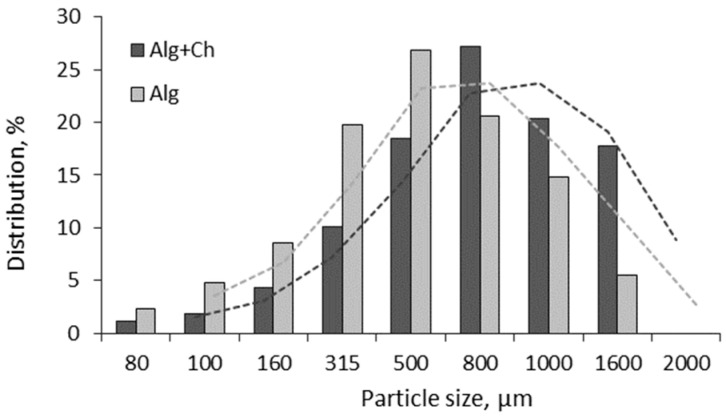
Particle size distribution of the freeze-dried alginate microcapsules. Samples: Alg—alginate, Alg+Ch—alginate–chitosan. Dashed lines—average trend lines.

The particle sizes of both microcapsule formulations loaded with *L. paracasei* cells in the dry form were distributed mainly between 80–160 μm (2.4–8.6%), 315 μm (19.8%), 500–800 μm (26.8–20.5%), and 1000–1600 μm (14.8–5.2%) in the case of alginate; and between 80–160 μm (1.2–4.3%), 315 μm (10.1%), 500–1000 μm (18.4–27.2%), and 1600 μm (17.7%) in the case of alginate–chitosan microcapsules (Figure 3).

These findings align with those reported by Albadran et al., who observed that alginate–chitosan capsules exhibited a diameter of approximately 2 mm before drying, which reduced to 0.98 and 1.34 mm post-drying [32]. Similarly, Fareez et al. prepared capsules with sizes ranging from 1312.4 to 1335.7 μm [33]. In contrast, Chavarri et al. developed alginate and alginate–psyllium capsules with sizes ranging from 345.43 to 542 μm using extrusion techniques and freeze-drying [34]. These variations in bead sizes can likely be attributed to differences in polymer concentration and composition [13].

Incorporating microencapsulated bacterial cells into food products represents an innovative approach to functional foods. However, significant challenges persist in the microencapsulation of probiotic cells, as the essential characteristics of microcapsules based on immobilized cell technology may conflict with the requirements for their use in food products. The application of encapsulated probiotics in the food industry is particularly challenging due to the relatively large size of bacterial cells (typically 1–4 μm), which can result in the formation of large capsules, potentially compromising the sensory qualities of foods [35,36]. Fareez et al. demonstrated that larger microcapsules provide enhanced protection for encapsulated cells in harsh environments [33]. Conversely, Martin et al. [37] and Heidebach et al. [27] reported that for food applications, capsule sizes should ideally not exceed 100 μm to avoid adverse sensory effects on the final product.

In our study, the size of freeze-dried alginate and alginate-coated chitosan microcapsules containing immobilized bacterial cells ranged from 80 µm to 1.6 mm (Figure 3), indicating that the microcapsules produced are sufficiently large to ensure effective cell loading and protection against environmental conditions, thereby confirming the potential of immobilized cell technology-based microcapsules for use in the food production industry.

### 2.2. Survivability of L. paracasei Cells Microencapsulated in Alginate Gel Matrix during Storage

#### 2.2.1. Bacterial Cell Stability during Storage of Wet Capsules

**Figure 4:**
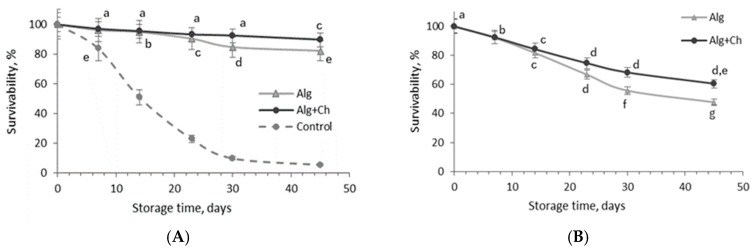
Survivability of *L. paracasei* gels encapsulated in wet alginate (Alg) and alginate–chitosan (Alg+Ch) capsules during 45-day storage at 4 °C (**A**) and 20 °C (**B**) temperatures. Control—free *L. paracasei* cells. Mean values within a line with different letters are significantly different (*p* < 0.05).

For the evaluation of LAB stability during refrigeration and storage at ambient temperature that may simulate the potential stress for cells during food storage, the count of the encapsulated *L. paracasei* cells was determined during 45-day storage of wet capsules at 4 °C and 20 °C temperatures (Figure 4).

The survivability of free *L. paracasei* cells after 14, 30, and 45 days of storage at 4 °C temperature was 51.1%, 9.9%, and 5.4%, respectively, of the initial number (1.5 × 10^9^ CFU/mL) (Figure 4A). The results showed that bacterial cells loaded in alginate or alginate coated-chitosan microcapsules demonstrated higher stability during storage compared to free cell culture. After two weeks of storage, in both cases, bacteria still maintained 95% survivability. The count of *L. paracasei* cells encapsulated in alginate and alginate–chitosan after 24 days of storage at 4 °C temperature was 90.34 and 93.11%, respectively, from the initial count (1.38 × 10^10^ and 1.02 × 10^10^ CFU/g, respectively), while a more prominent reduction in bacterial cells (84.62 and 82.05%) was fixed in the alginate capsules after 30 and 45 days, respectively.

In the case of *L. paracasei* survivability in double-coated capsules during 30–45 day storage periods, 92.31 and 89.51% of bacterial cells, respectively, survived in alginate–chitosan microcapsules (Figure 4A). Results indicate that the influence of the polymers used for the encapsulation of probiotics on their stability became evident.

The experiment of storage at room temperature showed that storage parameters, temperature, and time had a significant effect on the encapsulated bacteria count, and, in particular, it was clearly fixed already after 14 days of storage (Figure 4B). Furthermore, after 14, 30, and 45 days of storage at 20 °C temperature, the alginate and alginate–chitosan microcapsules contained 81.66 and 83.32%, 55.62 and 68.13%, and 47.53 and 60.34% bacteria from the initial count, indicating that chitosan-coated alginate microcapsules can ensure greater bacterial cell stability when microcapsules are stored in wet form (for example liquid sourdough) at both temperatures.

In studying the survivability of microencapsulated *L. paracasei* cells under different storage temperatures, it was demonstrated that temperatures of 4 °C and 20 °C did not appear to be harmful to the dried encapsulated cells. This observation can be attributed to the fact that bacterial viability was less dependent on the matrix used and more strongly influenced by the water activity of the dried capsule matrix [38]. It has been reported that the viability of *L. rhamnosus* GG in a flaxseed matrix was reduced by only 0.29 log units at an a_w_ of 0.11, while at an a_w_ of 0.22, the reduction was already 2.4 log units during storage at room temperature for up to 14 months.

According to Trabelsi et al. [12], encapsulating *Lactiplantibacillus plantarum* TN8 in alginate coated with chitosan was effective in maintaining the stability of the probiotic bacteria during 8 weeks of storage at 4 °C, with cell viability decreasing from 99.35% after 30 days to 96.14% after 60 days. In contrast, for alginate microspheres, viability decreased from 97.4 to 74%, indicating that the addition of chitosan as a coating material enhanced the viability of the microspheres [12]. The chitosan coating likely contributes to this stability by reducing the permeability of the alginate’s porous structure, thus limiting water ingress and protecting the encapsulated cells from external stresses [30]. This mechanism aligns with the observed decrease in cell viability over time. The more gradual decline in viability in the chitosan-coated capsules suggests that the coating provides an additional protective barrier, slowing the degradation process.

Thus, by reducing permeability and controlling moisture ingress, chitosan-coated alginate encapsulation enhances the survival of probiotic bacteria during storage. Taken together, selecting appropriate encapsulating materials and regulating water activity to a low value may offer promising strategies for extending the shelf life of dried probiotic products.

#### 2.2.2. The Survivability of Bacteria during Storage of Freeze-Dried Alginate-Based Microcapsules

**Figure 5:**
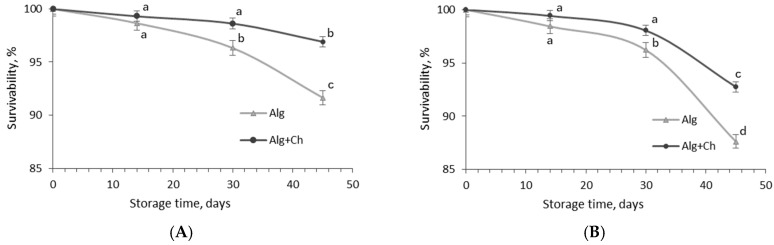
Survivability of *L. paracasei* during storage at temperatures of 4 °C (**A**) and 20 °C (**B**) of freeze-dried alginate (Alg) and alginate–chitosan (Alg+Ch) gel microcapsules. Mean values within a line with different letters are significantly different (*p* < 0.05).

The results of the survivability testing of encapsulated bacteria during storage of freeze-dried microcapsules at temperatures of 4 °C and 20 °C are presented in Figure 5. The chitosan coating showed a significant influence (*p* < 0.05) on the survival of microorganisms in dried microcapsules during storage at 4 °C (Figure 5A). It was determined that 96.3 and 91.6%, and also 98.6 and 96.9% of cells, respectively, survived in alginate and alginate–chitosan microcapsules after 30 and 45 days of storage at 4 °C. Although ambiguous results were obtained, it should be emphasized that, in both cases, the number of LAB, at around 1.50 × 10^9^–2.96 × 10^9^ CFU/g, remained after 30–45 days of storage in both types of freeze-dried microcapsules.

Storage at ambient conditions (20 °C) significantly (*p* < 0.05) affected the number of encapsulated bacteria. The survivability of *L. paracasei* encapsulated in alginate and alginate–chitosan gels was 98.4 and 99.4%, respectively, on the 14th day of storage, and it decreased to 96.2 and 98%, respectively, on the 30th day, and further, to 87.6 and 92.8%, respectively, at the end of storage (Figure 5B).

These results indicate that freeze-drying the microcapsules allowed for the retention of a higher number of viable *L. paracasei* cells compared to storing the capsules in a wet form. Although alginate capsules are insoluble in water, they are chemically sensitive to monovalent Ca^+^ ions and low pH, which can weaken the capsule walls and increase their permeability [39]. Therefore, coating alginate microspheres with chitosan can help address issues related to permeability and mechanical fragility [40].

### 2.3. Fermentation Ability of Free and Microencapsulated L. paracasei Bacteria

According to the literature, *L. paracasei* bacteria are acid-tolerant facultative heterofermentative microorganisms producing lactic acid as the main product of hexose metabolism, while lactate and acetate can be produced from pentoses under certain conditions or from specific substrates [41]. In our previous studies, *L. paracasei* was able to provide considerable growth in rice polish medium with an optimal temperature range of 30–37 °C [42], fermenting mainly D-ribose, D-galactose, D-glucose, D-fructose, D-mannose, and L-rhamnose [43].

The rice sourdough fermentation ability of *L. paracasei* at temperatures of 30 and 35 °C was evaluated based on the analysis of changes in pH and total titratable acidity (TTA), and also, in the number of bacterial cells. The liquid sourdough was prepared using free bacterial cells (initial bacterial count in the rice/flour medium was 7.15 log_10_ CFU/g) (Table 2).

The results indicated the largest reductions in pH values, averaging 17.6 and 14.0%, and the most significant increases in TTA values, ranging from 91.5 to 98.8% and 62.4 to 67.1%, were observed during 6–12 h and 12–24 h of fermentation, respectively, depending on the processing temperature.

During the final 12 h of fermentation, the acidity of sourdough was significantly lowered, with an average pH reduction of 4.9% and TTA values increasing by an average of 11.6%. Processing at a temperature of 35 °C resulted in an average 12.8% increase in TTA values. TTA values of 4–5 °N for wheat bread sourdough can be achieved within 24 h of fermentation with *L. paracasei*.

An analysis of the changes in LAB count during sourdough fermentation confirmed the impact of process temperature and time on fermentation efficiency (Table 3). A notable increase in bacterial count, averaging 13.6%, was observed during 12–24 h of the processing period. Increasing the sourdough fermentation temperature from 30 °C to 35 °C led to an increase of 0.2–0.3 log_10_ CFU/g in *L. paracasei* cell count during the 24–36 h period (Table 1). The results indicate that using a temperature of 35 °C for sourdough fermentation with pure *L. paracasei* allows the processing time to be shortened to 24 h instead of 36 h, yielding a sourdough pH of 3.6 and a bacterial cell count of approximately 10^8^ CFU/g.

The results of analysis of changes in acidity during sourdough fermentation with encapsulated *L. paracasei* bacteria are presented in Table 3. The experiment demonstrates the promising potential of encapsulated bacteria for repeated use in sourdough fermentation. When using encapsulated bacteria, the sourdough’s acidity increased most significantly (an average 3.3-fold increase in TTA values) between the 12th and 24th hours of fermentation at both tested temperatures (Table 3). In contrast, the greatest increase in acidity for sourdough prepared with free *L. paracasei* bacteria occurred during the first 12 h of processing (Table 3). During the final 12 h of fermentation, the increase in sourdough TTA values was less pronounced (58.2–66.7%).

A similar trend was observed in the pH changes (Table 3), showing the chitosan coating having a significant effect (*p* < 0.05). Chitosan coated capsules slowed down the organic acid formation in sourdough; the reduction in pH values for sourdough prepared with *L. paracasei* encapsulated in chitosan–alginate was 2–4% lower compared to those encapsulated in alginate alone. These findings suggest that the chitosan coating acted as a barrier, while the alginate-encapsulated lactic acid bacteria were gradually released into the sourdough.

The second fermentation cycle demonstrated a more intensive sourdough acidification, with a statistically significant dependence on both temperature and fermentation duration (*p* < 0.05) (Figure S1). The pH values of the sourdough were lower than those observed in the first fermentation cycle, ranging between 3.73 and 4.04 for alginate and between 3.64 and 4.05 for alginate–chitosan, depending on the capsule amount. The most pronounced pH changes at 30 °C fermentation (12.8–13.0% for alginate and 10.3–11.9% for alginate–chitosan) occurred within the 0–6 h and 6–12 h intervals, in contrast to the first fermentation cycle (Table 3). At 35 °C, the sourdough acidity increased more sharply during the first 6 h (16–17% for alginate and 13–14% for alginate–chitosan), followed by changes during the 6–12 h period (12.7% for alginate and 13.5–14.7% for alginate–chitosan). During the later stages of fermentation (12–36 h), the percentage reduction in pH ranged from 5.0 to 8.4%, depending on the temperature. A similar trend was observed for total titratable acidity (TTA), further confirming that the second fermentation cycle was more intensive compared to the first, enabling the production of higher quantities of organic acids in a shorter time (Figure S1).

Based on the experimental results, it can be concluded that the effectiveness of the wet microcapsules slightly decreased upon secondary use (Figure S1), however, the acidity of the sourdough remained proper for the preparation of baked goods. During the first fermentation cycle, the chitosan coating served a barrier function, allowing the encapsulated *L. paracasei* bacteria to be steadily released into the sourdough. In the case of secondary use, it was observed that the effectiveness of bacteria encapsulated in pure alginate was slightly reduced compared to the alginate–chitosan capsules. This may be attributed to a higher release of lactic acid bacteria into the sourdough during the initial stage [13], resulting in lower TTA values during repeated sourdough fermentation.

These findings can be explained by the influence of pH conditions and lactic acid on ionic carbohydrates. Under acidic conditions, such as those in sourdough fermentation, organic acids reduce the pH, which can affect the ionization state of alginate [44]. Specifically, the decrease in pH leads to the protonation of alginate, weakening its ionic bonds with Ca ions and reducing the gel structural integrity, thus, leading to a breakdown in the encapsulating matrix [45]. However, chitosan, a cationic polysaccharide, forms electrostatic interactions with the negatively charged alginate molecules and helps stabilize the microcapsules by providing a protective layer [46]. The presence of chitosan reinforces the overall stability of the encapsulation system even when exposed to the low pH caused by lactic acid.

In addition, the unique properties of alginate and chitosan contribute to the stability and survival of probiotic bacteria. Alginate’s gel-forming ability helps retain moisture within the encapsulated environment, providing a protective barrier against desiccation and environmental stressors that could compromise bacterial viability [47]. Meanwhile, chitosan is known for its antimicrobial properties, which can inhibit the growth of harmful bacteria, further supporting the viability of probiotic strains in food matrices.

### 2.4. The Influence of Sourdough and Bacteria Microencapsulated in Alginate Gel Matrix on the Quality and Acceptability of Baked Goods

The influence of different sourdoughs (liquid traditional and dried sourdough) and lyophilized *L. paracasei* alginate and alginate–chitosan microcapsules on the quality of baked goods was evaluated by changes in the shape retention index (*I_h_*), specific volume, total titratable acidity (TTA), and LAB count in bread crumb after baking (Table 4). Photo images of the tested baked goods with different sourdoughs are presented in Figure S2.

The baked good prepared with *L. paracasei*-fermented sourdoughs and also with lyophilized *L. paracasei* alginate microcapsules had higher specific volumes by 67–75.4%, compared to the control sample (1.91 cm^3^/g), with the highest values for bread (3.35 cm^3^/g) prepared with liquid sourdough (sample SR), while the addition of lyophilized alginate–chitosan capsules increased the specific volume of bread by a lower amount (41.9%) compared to the control buns.

In this case, the encapsulated bacteria were less effective compared to the natural sourdoughs, possibly due to the relatively short dough fermentation time (30 min). Notably, all breads had *I_h_* values ≥ 0.55 (Table 4), indicating well-shaped baked goods (Figure S2). It should be noted that although the control bread had an only slightly higher *I_h_* value compared to the bread prepared with lyophilized sourdough, the latter exhibited significantly higher crumb porosity, similar to the breads with lyophilized *L. paracasei* in alginate and alginate–chitosan capsules.

The TTA values of the tested baked goods varied between 0.9 and 3.4 °N (Table 4). The use of sourdough or microencapsulated *L. paracasei* bacteria led to an 18% increase in bread crumb acidity (sample Alg+Ch) and up to a 3.8-fold increase (sample SR), respectively. However, the TTA values of the crumb in bread prepared with lyophilized alginate and alginate–chitosan capsules were found to be 60 and 70% lower, respectively, than that of the sample prepared with traditional sourdough.

The sourdough influences not only the quality parameters of the bread, such as acidity and texture, but also its overall acceptability [48,49]. The buns made with traditional sourdough were rated as highly acceptable, while those with encapsulated bacteria were evaluated as less acceptable, though they still showed significantly higher acceptability compared to the control samples (Table 4).

When directly comparing the survival of encapsulated and free bacterial cells within the same type of sourdough, it was found that the reduction in bacterial count was smaller for encapsulated *L. paracasei* cells than for free bacteria in the same dough. Specifically, in buns with freeze-dried encapsulated bacteria, the reduction in cell numbers were 1.06 and 1.23 log_10_ CFU/g, whereas in buns made with traditional sourdough (sample SR) containing free bacterial cells, the reduction was more significant, with a drop of approximately 2.46 log_10_ CFU/g after baking. This comparison highlights the protective effect of encapsulation, which helps maintain a higher viability of bacterial cells during the baking process, as seen in the higher final bacterial count in buns with encapsulated bacteria (6.36–6.51 log_10_ CFU/g) compared to those with traditional sourdough (5.27 log_10_ CFU/g). Additionally, the use of a relatively lower baking temperature (180 °C) likely contributed to the overall survival of bacterial cells, both free and encapsulated.

Temperatures above 45 °C are known to be critical for probiotics in free form and to lead to a decrease in survival depending on the processing temperature [26]. In the study by Zhang et al. [16], the baking process significantly reduced the viability of free *Lactobacillus plantarum* cells to approximately 10^4^–10^5^ CFU/g in bread (dough weight 5–60 g), which was baked at temperatures of 175–235 °C for 8 min. Zhang and co-authors [50] presented a kinetic model describing the inactivation of bacteria during isothermal heating at 90 °C and baking. They found that survival of freeze-dried bacteria during heat treatment depended on the physical properties of the encapsulating materials, as well as on the thermo-resistance of the bacterial strain and the combination of natural polymers used for encapsulation.

Seyedain-Ardabili et al. produced symbiotic sourdough bread prepared with *Lactobacillus acidophilus* LA-5 and *L. casei* 431 encapsulated in calcium alginate and Hi-maize resistant starch via an emulsion technique, then coated with chitosan [51]. *L. casei* 431 demonstrated greater resistance to high temperatures compared to *L. acidophilus* LA-5, with significantly higher bacterial cell counts observed when the alginate and Hi-maize starch microcapsules coated with chitosan were used for breadmaking. Different carrier combinations, such as alginate–maltodextrin and alginate–xanthan gum, provided different abilities in protecting *L. acidophilus* bacteria, with >80% survivability under different temperatures, while the viability of free *L. acidophilus* cells was only 32.9% [52].

In our study, we observed similar patterns, where the encapsulated bacteria demonstrated significantly higher survival rates during baking. Specifically, buns prepared with encapsulated *L. paracasei* exhibited a smaller reduction in bacterial counts compared to those with free bacteria in traditional sourdough, where the reduction was more pronounced after baking. These results clearly show that encapsulation can substantially enhance the thermal resistance of LAB during baking. By forming a protective barrier around bacterial cells, encapsulation with alginate and chitosan can help maintain the viability of LAB, ensuring that baked goods meet the standard criteria for probiotic products with counts above 10^6^ CFU/g. In contrast, free bacteria, without this protective encapsulation, suffer greater thermal inactivation, reducing their viability and limiting the probiotic potential of the final product.

Thus, the use of microencapsulation, particularly with dry alginate-encapsulated LAB, is an effective strategy for improving the stability of probiotics in functional bread production, ensuring sufficient numbers of viable microorganisms even after exposure to high temperatures during baking.

### 2.5. The Influence of Sourdoughs and Microencapsulated Bacteria Additives on Bread Shelf Life during Storage

The molding of wheat–rice flour buns was monitored over 10 days of storage (Table 5). Mold formation on the surface of the control buns was observed after 4 days.

The experiment showed that the use of encapsulated *L. paracasei* bacteria powder additives prolonged the shelf life of wheat buns, delaying mold growth by 3 days compared to the controls. In the case of bread products with liquid and dried *L. paracasei* sourdoughs, molding was noticed two days later as compared to the control. After 7 days of storage, the control sample was completely molded, the sample with liquid sourdough had a few visible mold colonies, while the buns with encapsulated bacteria and dry sourdough only began to mold. After 10 days of storage all samples were completely molded.

There may be a significant relationship between the number of viable bacteria in the bread and bread molding (Table 4). The possibility of extending the shelf life of bread by using sourdough was reported by Denkova and co-authors [53]. However, a high sourdough content, due to increased acidification and the activity of amylolytic and proteolytic enzymes, can negatively affect the porosity, specific volume, and overall acceptability of bread. In our study, the application of microencapsulated bacteria did not slow down the molding of baked goods as effectively as the 10% *L. paracasei*-fermented rice flour additives; however, it significantly more effectively maintained a high count of viable cells in the baked goods. Moreover, it should be noted that mold inhibition and the probiotic effect strongly depend on the antimicrobial properties and resistance of the LAB strain, and, of course, on the amount of bacteria added [54]. Thus, microencapsulation’s effects of increasing the viability of probiotic cells in bakery products to either protect them against disturbing conditions or assure their stability under storage conditions during the product’s shelf life can be characterized as the most useful.

## 3. Conclusions

The encapsulation efficiency of *L. paracasei* in alginate was slightly higher compared to microcapsules coated with chitosan, likely due to the relatively greater amount of polymeric materials. Stabilization of the encapsulated bacteria using freeze-drying slightly affected the number of bacteria; however, it was a more effective method for maintaining bacteria viability during storage than keeping the capsules in a wet state. Encapsulated bacteria showed eligible fermentation ability, compared to traditional sourdoughs, while double gel-coated cells exhibited a slower acidification during sourdough fermentation, but maintained higher efficiency in the second fermentation cycle.

These findings have practical significance, offering a potential improvement in sourdough production, where microbial cultures could be reused while maintaining the efficiency of the fermentation process. This could lead to more consistent product quality and improved control over fermentation outcomes in industrial applications. Furthermore, the microencapsulated bacteria had a positive effect on bread quality and significantly extended its shelf life compared to traditional sourdoughs. Encapsulation in a calcium alginate gel matrix led to preserving lactic acid bacteria during bread baking.

Regarding future perspectives, an appropriate amount of dried *L. paracasei* alginate or alginate–chitosan microcapsules can be recommended for the production of baked goods of acceptable quality and sensory properties with a viable lactic acid bacteria count of around 10^6^ CFU/g in crumb, enabling wheat baked goods to meet the standard criteria for probiotic products.

## 4. Materials and Methods

### 4.1. Bacterial Strain

The lactic acid bacteria strain *Lacticaseibacillus paracasei* (NBRC 15889), provided by the Veterinary Academy of the Lithuanian University of Health Sciences (Kaunas, Lithuania) and characterized as an acid-tolerant strain [42], was used for the encapsulation and fermentation experiments. *L. paracasei* showed good stability at pH 2.5 (after 3 h of incubation retained 88.6% of viability) and growth at temperatures between 30 and 37 °C. Prior to each of the experiments, the bacteria were propagated in an MRS broth (Oxoid Ltd., Basingstoke, UK) at 35 °C for 48 h under anaerobic conditions. The obtained *L. paracasei* cell suspension (10.35 log_10_ CFU/mL) was used for the encapsulation purpose.

### 4.2. Materials

The natural polymers—sodium alginate (Cas No. 9005-38-3, Merck KGaA, Darmstadt, Germany) and chitosan (Cas No. 9012-76-4, Merck KGaA, Darmstadt, Germany)—were used for the encapsulation of bacterial cells. Wheat flour (type 550, protein 12%, fat 1.3%, moisture 14.5%), obtained from Kauno Grudai Ltd. (Kaunas, Lithuania), rice flour (protein 5.96%, fat 1.4%, moisture 14.5%) (SC “Ūstukių malūnas”, Pasvalys, Lithuania), baker yeast, and salt purchased from the local market were used for wheat breadmaking experiments.

### 4.3. Encapsulation of Bacterial Cells

#### 4.3.1. Preparation of the Encapsulation Solutions

Encapsulation of *L. paracasei* cells was performed by the extrusion method according to Abbaszadeh et al. [4]. Glass containers and reagents were autoclaved for 15 min at 121 °C. Sodium alginate solution 4% (*w*/*v*) was prepared in distilled water. Chitosan solution (4 g/L) was prepared in distilled water acidified with glacial acetic acid. The pH of the solution was adjusted to 5.8 by 1 M NaOH solution. Ca-lactate was used as a cross-linker for the encapsulation via the extrusion method to increase encapsulation efficiency [55].

#### 4.3.2. Encapsulation Procedure

For the encapsulation, 30 mL of *L. paracasei* cell suspension (10.35 log_10_ CFU/mL) was mixed with 30 mL of sterile sodium alginate solution. The mixture was then extruded dropwise into a sterile 0.1 M calcium lactate solution (200 mL) with a magnetic stirrer (150 rpm). A common micropipette (2–200 μL) tip (0.45 mm inner diameter) was used to produce the capsules. Alginate–bacterial cell capsules were left to cross-link for 30 min, after which capsules were washed twice with sterile water, collected onto Whatman filter paper to remove excess of water, then kept for 40 min in chitosan solution (200 mL) under gentle stirring (100 rpm/min). The obtained alginate capsules coated with chitosan were washed with sterile water and collected as described above. Microcapsules were stored in the refrigerator until further use. Some of the wet capsules were subjected to freeze-drying. Encapsulation efficiency (EE) was calculated as the ratio of the logarithms of the number of bacterial cells loaded in microcapsules to the number of bacterial cells in the encapsulation solution [13], with each encapsulation process performed in triplicate.

### 4.4. Freeze-Drying

The liquid sourdough or wet microcapsules were frozen at −20 °C overnight and then freeze-dried (vacuum pressure 0.12 mbar; condensing temperature −30 °C) using a freeze dryer (MAXI DRY LYO, Gemini B.V., Apeldoorn, The Netherlands). The processing time was 24 and 42 h for microcapsules and liquid sourdough, respectively.

### 4.5. Microcapsule Morphology and Particle Size Analysis

Alginate and alginate–chitosan microcapsule images were captured using a photo camera. An average microcapsule size (mm) was measured using a standard caliper (accuracy ± 0.02 mm) by selecting 50 capsules of each test batch and calculating mean diameter values. An optical microscope (Axio Imager.Z2, Carl Zeiss Oy, Vantaa, Finland; magnification 100×) was used to observe and record microcapsules after freeze-drying. The freeze-dried sample (each 1 g) for optical microscopy was used without additional preparation. Analysis of the particle size distribution of dried microcapsules was carried out in a laboratory sieve shaker equipped with an electromagnetic drive (HAVER EML 200 Pure, VWR International, LLC, Ismaning, Germany). The raw material (100 ± 0.001 g) was separated using 9 screens with pore diameters between 0.08 and 2.0 mm.

### 4.6. Evaluation of the Survivability of the Microencapsulated Bacteria under Simulated Processing Conditions

#### 4.6.1. Sourdough Fermentation

The sourdough samples were prepared by mixing 100 g of rice flour and 250 mL of water with a further addition of 5 mL of *L. paracasei* cell suspension (1.5 × 10^9^ CFU/mL) or bacterial cells encapsulated in alginate and chitosan-coated alginate microcapsules (10% of flour weight). The sourdoughs were subjected to 36 h fermentation at temperatures of 30 °C and 35 °C. The samples for acidity and lactic acid bacteria count analysis were collected at the initial time point and after 6, 12, 24, and 36 h of fermentation.

#### 4.6.2. Storage of Microcapsules

The viability of the encapsulated bacteria in wet and freeze-dried capsule form was evaluated during storage at temperatures of 4 °C and 20 °C. Each sample of wet microcapsules (sample weight 10 g) was placed in a tightly closed glass bottle and stored for 45 days in the refrigerator to model long-term storage conditions. Each sample (10 g) of dried microcapsules was placed in a tightly closed plastic bag and stored for 45 days at room temperature. Test samples (1 g) were taken after 7, 14, 24, and 30 days, and at the end of storage. The survivability (survival rate) of the encapsulated cells was calculated as the percentage ratio of the initial count of viable cells (CFU/g) and the count of cells at the selected storage time point.

### 4.7. Determination of pH and Total Titratable Acidity

The pH values were measured directly by a pH electrode (PP-15, Sartorius AG, Göttingen, Germany). For the titratable acidity (TTA) determination, a 10 g sourdough sample was homogenized with 90 mL of distilled water. The TTA was determined according to the AACC 02-31.01 method [56], and was expressed in Neiman degrees (°N), where 1 °N is the volume in milliliters of 1 M NaOH required to neutralize organic acids in 100 g of a sample.

### 4.8. Breadmaking Procedure

The test wheat buns were made using liquid or freeze-dried *L. paracasei* sourdoughs and encapsulated bacteria according to the recipes and technological process parameters presented in Table S1. The sourdough fermentation was performed at a temperature of 35 °C until the pH reached 3.6–3.8 (bacterial count 8.48 log_10_ CFU/g). Part of the sourdough was freeze-dried (bacterial count 7.53 log_10_ CFU/g) and also used in the breadmaking experiment.

### 4.9. Determination of the Total Number of Lactic Acid Bacteria

The number of viable bacterial cells in liquid samples was determined using the plate count method on MRS agar (CM0361, Oxoid, Basingstoke, UK) under anaerobic conditions according to ISO 15214 [57] recommendations. One milliliter of bacterial suspension was mixed with 9 mL of sterilized distilled water and, after appropriate dilutions (up to 10^−4^–10^−8^), cells were plated on MRS agar (Oxoid Ltd., Basingstoke, UK) and incubated at 30 °C for 72 h under anaerobic conditions. Enumeration of microencapsulated cells was performed according to Chavarri et al. [34]. One gram of the microcapsules was dissolved in 9 mL of sterile distilled water and homogenized until complete dissolution. The samples were serially diluted to the appropriate concentration using 0.1% (*w*/*v*) peptone and the viable cells were determined by the spread plate method using MRS agar. After incubation at 30 °C for 72 h, the formed cell colonies were calculated, and the viable cell number was expressed as a log of colony forming units per milliliter (log_10_ CFU/mL) of sample or per gram of microcapsules (log_10_ CFU/g).

### 4.10. Determination of Bread Quality

The loaf volume was determined according to the AACC-approved Method 10-05.01 (rapeseed displacement test) [56] by measuring the volume of rapeseed displaced by the bread loaf. The specific volume was calculated by dividing the loaf volume by the loaf weight and expressed for 1 g of bread crumb. Bread crumb porosity was ascertained according to the Lithuanian standard method [58]. The bread shape retention index (*I_h_* = *h*/*l*) was calculated by dividing the height (*h*) and the length (*l*) of the bun slice measured in mm. The acceptability of baked goods was determined according to the ISO 8586-1 method [59] by 18 evaluators for preliminary sensory acceptability using a 10 mm hedonic line scale ranging from 10 (extremely like) to 0 (extremely dislike) for each sensory characteristic. Before analysis, each bun was sliced and divided into 20 × 20 × 10 mm probes without the crust. Sensory evaluation was performed 24 h after baking.

#### 4.10.1. The Viability of Lactic Acid Bacteria in Baked Goods

The number of LAB cells remaining in the bread was determined 18 h after baking and storage at room temperature according to Seyedain-Ardabili et al. [51]. For the analysis, 10 g of bread crumb was homogenized with 90 mL of phosphate buffer (0.1 M, pH 7.0), then mixed for 10 min with a thermomixer (IKA-Werke GmbH & Co., Staufen in Breisgau, Germany). Since the chitosan-coated capsules are insoluble in phosphate buffer, they were homogenized with 90 mL of citrate buffer (0.1 M, pH 6.2) and mixed until dissolving. The number of lactic acid bacteria was determined by plating on MRS agar according to the description presented in Section 2.4.

#### 4.10.2. Evaluation of Bread Molding during Storage

The mold formation on surface-coated bread prepared with different LAB sourdoughs, was monitored during 10 days of storage at 20 °C temperature and 70% RH conditions. For the analysis of molding, bun slices of each sample in duplicate were packed in plastic containers and stored in a controlled environment chamber. The surface of the bun slices was monitored daily for visible fungi colonies. Molding intensity was identified as (−) no visible colonies, (+) one–two colonies (diameter 1–2 mm), (++) few visible colonies (diameter 3–5 mm), and (+++) pronounced intensive mold growth (diameter > 6 mm).

### 4.11. Statistical Analysis

All chemical analyses were performed at least in triplicates. The encapsulation experiment was repeated tree times. Data analysis was performed using the Microsoft Office Excel 2016 (ver. 24.08) software package. All chemical analysis and fermentation and encapsulation experiments were repeated three times, and baking experiments and bread quality analysis were performed in duplicate. One-way analysis of variance (ANOVA) was used to estimate the significant differences between data groups. The significance level of the factor was determined according to Fisher’s (F) test at a confidence level of 95%.

## Figures and Tables

**Table 1:** The encapsulation efficiency and logarithmic counts of bacterial cells in wet and freeze-dried microcapsules.

Coating	LAB Count in Wet Microcapsules, log_10_ CFU/g	EE *, %	Microcapsule Size, mm	LAB Count in Freeze-Dried Microcapsules, log_10_ CFU/g
Alginate	10.14 ± 0.01 ^a^	97.97 ^a^	2.8 ± 0.2 ^b^	9.50 ± 0.05 ^a^
Alginate–chitosan	10.01 ± 0.03 ^a^	96.71 ^a^	3.7 ± 0.3 ^a^	9.19 ± 0.03 ^a^

Data are expressed as mean values (*n* = 3) ± SD. Mean values within a column with different letters are significantly different (*p* < 0.05). * Encapsulation efficiency. LAB—lactic acid bacteria.

**Table 2:** The pH, total titratable acidity (TTA) values, and *L. paracasei* bacteria count in rice sourdough fermented at different temperatures.

Fermentation Time, h	pH		TTA		LAB Count, log_10_ CFU/g	
	30 °C	35 °C	30 °C	35 °C	30 °C	35 °C
0	5.70 ± 0.01 ^a^	5.71 ± 0.01 ^a^	1.13 ± 0.01 ^h^	1.12 ± 0.02 ^h^	7.15 ± 0.11 ^b^	7.15 ± 0.11 ^b^
6	5.23 ± 0.01 ^b^	5.12 ± 0.00 ^b^	1.53 ± 0.01 ^g^	1.65 ± 0.02 ^f^	7.21 ± 0.21 ^b^	7.24 ± 0.33 ^b^
12	4.33 ± 0.01 ^c^	4.21 ± 0.02 ^d^	2.93 ± 0.02 ^e^	3.28 ± 0.01 ^e^	7.36 ± 0.17 ^b^	7.46 ± 0.15 ^b^
24	3.78 ± 0.02 ^e^	3.56 ± 0.02 ^f^	4.76 ± 0.01 ^d^	5.48 ± 0.02 ^b^	8.48 ± 0.05 ^a^	8.69 ± 0.22 ^a^
36	3.60 ± 0.0 ^e,f^	3.38 ± 0.01 ^g^	5.25 ± 0.02 ^c^	6.19 ± 0.02 ^a^	8.86 ± 0.13 ^a^	9.06 ± 0.14 ^a^

Data are expressed as mean ± SD (n = 3). Mean values in columns with different letters are significantly different (*p* < 0.05).

**Table 3:** The changes in pH and titratable acidity (TTA) of sourdough fermented with wet *L. paracasei* microcapsules at different temperatures.

Fermentation Time, h	30 °C		35 °C	
	Alg	Alg+Ch	Alg	Alg+Ch
pH				
0	5.88 ± 0.01 ^a^	5.87 ± 0.01 ^a^	5.88 ± 0.01 ^a^	5.87 ± 0.01 ^a^
6	5.70 ± 0.02 ^a^	5.76 ± 0.02 ^a^	5.43 ± 0.01 ^b^	5.50 ± 0.01 ^b^
12	5.12 ± 0.01 ^d^	5.21 ± 0.01 ^c^	4.77 ± 0.01 ^f^	4.86 ± 0.02 ^e^
24	4.54 ± 0.01 ^h^	4.68 ± 0.01 ^g^	4.12 ± 0.01 ^m^	4.23 ± 0.01 ^k^
36	4.36 ± 0.01 ^i^	4.52 ± 0.01 ^h^	3.96 ± 0.01 ^n^	4.07 ± 0.01 ^m^
**TTA, °N**				
0	0.45 ± 0.01 ^p^	0.44 ± 0.01 ^p^	0.45 ± 0.01 ^p^	0.44 ± 0.01 ^p^
6	0.68 ± 0.01 ^m^	0.63 ± 0.01 ^n^	0.70 ± 0.01 ^m^	0.65 ± 0.01 ^n^
12	1.05 ± 0.01 ^k^	0.96 ± 0.01 ^l^	1.13 ± 0.02 ^h^	1.02 ± 0.01 ^k^
24	3.51 ± 0.02 ^e^	3.21 ± 0.01 ^g^	3.78 ± 0.01 ^d^	3.38 ± 0.01 ^f^
36	5.85 ± 0.01 ^b^	5.32 ± 0.02 ^c^	5.98 ± 0.01 ^a^	5.45 ± 0.02 ^b^

Data are expressed as mean ± SD (n = 3). Mean values in columns within the test groups with different letters are significantly different (*p* < 0.05). Alg—alginate microcapsules; Alg+Ch—alginate–chitosan microcapsules.

**Table 4:** The bread quality parameters and *L. paracasei* cell numbers in the dough and bread crumb after baking.

Bread Samples	Bread Quality Attributes					LAB Count, log_10_ CFU/g		
	Porosity, %	Specific Volume, cm^3^/g	*I_h_*	TTA, °N	Acceptability	Dough	Crumb	Reduction
K	67.64 ± 0.11 ^e^	1.91 ± 0.02 ^e^	0.64 ± 0.01 ^b^	0.90 ± 0.01 ^e^	4.2 ± 0.3 ^c^	-	-	-
SR	84.36 ± 0.12 ^a^	3.35 ± 0.03 ^a^	0.72 ± 0.03 ^a^	3.40 ± 0.01 ^a^	7.8 ± 0.4 ^a^	7.73 ± 0.21 ^a^	5.27 ± 0.11 ^b^	2.46
LSR	78.67 ± 0.06 ^b^	3.25 ± 0.02 ^b^	0.66 ± 0.02 ^b^	1.68 ± 0.01 ^b^	6.8 ± 0.2 ^b^	6.32 ± 0.14 ^b^	4.98 ± 0.05 ^b^	1.34
L_Alg_	74.42 ± 0.05 ^c^	3.19 ± 0.02 ^c^	0.58 ± 0.01 ^c^	1.20 ± 0.01 ^c^	6.5 ± 0.3 ^b^	7.59 ± 0.17 ^a^	6.36 ± 0.05 ^a^	1.23
L_Alg+Ch_	70.78 ± 0.02 ^d^	2.71 ± 0.04 ^d^	0.55 ± 0.01 ^d^	1.10 ± 0.01 ^d^	6.4 ± 0.5 ^b^	7.57 ± 0.23 ^a^	6.51 ± 0.21 ^a^	1.06

Data are expressed as mean values (n = 3) ± SD. Mean values within a column with different letters are significantly different (*p* < 0.05). K—wheat buns without sourdough; SR—buns with *L. paracasei*-fermented rice sourdough; LSR—buns with lyophilized rice sourdough; L_Alg_, L_Alg+Ch_—buns with lyophilized alginate or alginate–chitosan capsules; *I_h_*—shape retention index; TTA—total titratable acidity; LAB—lactic acid bacteria.

**Table 5:** The mold colony growth during storage of buns prepared with different sourdoughs.

Samples	4 Days	5 Days	6 Days	7 Days	8 Days	9 Days	10 Days
K	+	++	++	+++	+++	+++	+++
SR	−	−	+	++	++	+++	+++
LSR	−	−	+	+	++	++	+++
L_Alg_	−	−	−	+	+	++	+++
L_Alg+Ch_	−	−	−	+	++	++	+++

Mold growth intensity: (−) no visible colonies, (+) one–two colonies (diameter 1–2 mm), (++) three visible colonies (diameter 3–5 mm), and (+++) pronounced intensive mold growth (diameter > 10 mm). K—wheat buns without sourdough; SR—buns with *L. paracasei*-fermented rice sourdough; LSR—buns with lyophilized rice sourdough; L_Alg_, L_Alg+Ch_—buns with lyophilized alginate or alginate–chitosan capsules.

## Data Availability

The original contributions presented in the study are included in the article/Supplementary Material, further inquiries can be directed to the corresponding author.

## Supplementary Materials

The following supporting information can be downloaded at: https://www.mdpi.com/article/10.3390/gels10100641/s1, Table S1: The technological parameters and recipes of wheat buns produced with *L. paracasei* fermented sourdoughs or alginate (Alg) and alginate-chitosan (Alg+Ch) microcapsules; Figure S1: The changes in pH and titratable acidity during II sourdough fermentation with wet capsules of *L. paracasei* bacteria at different temperatures; Figure S2: Photo images of baked goods with different sourdough additives.
