# Supplementary material for: Exploring Calcium Alginate-Based Gels for Encapsulation of Lacticaseibacillus paracasei to Enhance Stability in Functional Breadmaking
Source: Gels. 2024 Oct 8;10(10):641. doi: 10.3390/gels10100641 (PMC11506860; doi:10.3390/gels10100641)
Supplement: Supplementary file 1 [file gels-10-00641-s001.zip › gels-3227243-supplementary.pdf]

Supplementary Materials

Table S1. The technological parameters and recipes of wheat buns produced with *L. paracasei* fermented sourdoughs or alginate (Alg) and alginate-chitosan (Alg+Ch) microcapsules

Recipe Components, g	Wheat Buns				
	Control	With Freeze-Dried Capsules		With Sourdough	
		Alg	Alg+Ch	Liquid	Freeze-dried
Wheat flour	135	135	135	135	135
Rice flour	15	15	15	-	-
Rice sourdough (moist. 70%)	-	-	-	42.75	-
Dried Alg capsules (moist. 3%)	-	3	-	-	-
Dried Alg+Ch capsules (moist. 3%)	-	-	6	-	-
Dried rice sourdough (moist. 4%)	-	-	-	-	15
Baker yeast (moist. 75%)	3.5	3.5	3.5	3.5	3.5
Salt (moist. 3%)	1.7	1.7	1.7	1.7	1.7
Dough yield (moist. 45%)	237.77	243.06	248.35	237.77	240.63

Technological process parameters	
Dough mixing	6 min; 26–28 °C
Dough fermentation	40 min; 30 °C; 80% RH
Dough roll weight	75 ± 2 g
Proofing	40 min; 35 °C; 80% RH
Baking	15 min; 180 °C

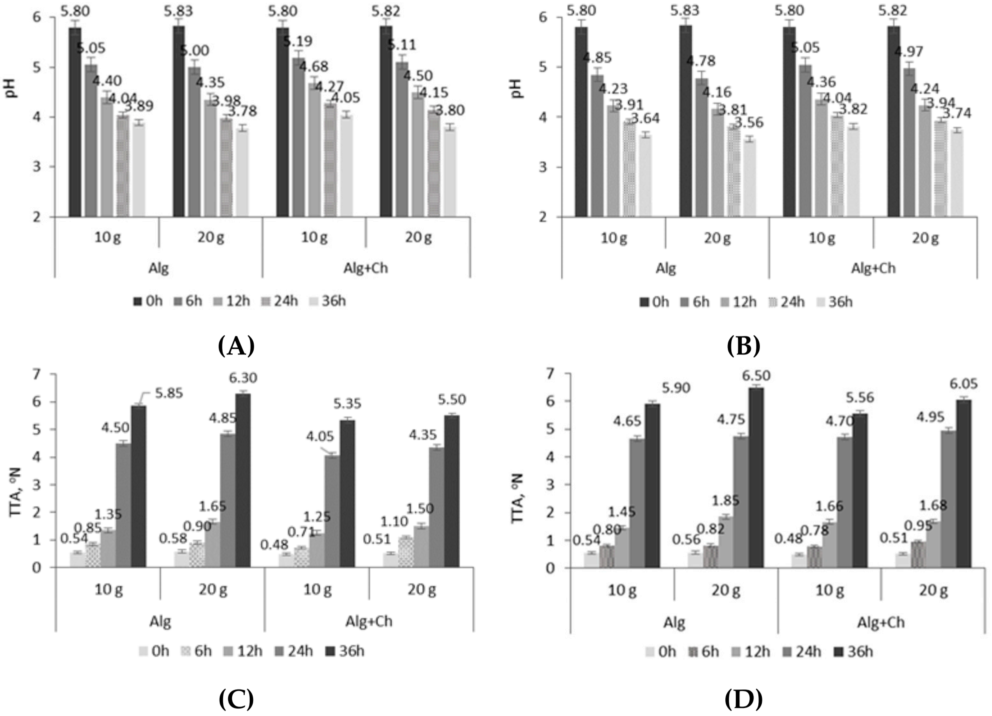

Figure S1. The changes in pH (**A, B**) and and titratable acidity (TTA) (**C, D**) during II cycle of sourdough fermentation with different amounts of wet capsulates of *L. paracasei* bacteria at different temperatures.

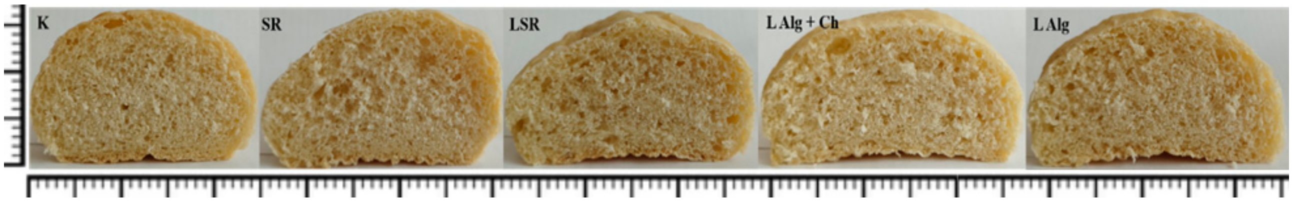

Figure S2. Photo images of baked goods with different sourdough additives. Bread samples: K – wheat buns without sourdough; SR – buns with *L. paracasei* fermented rice sourdough; LSR – buns with lyophilised rice sourdough; L_{Alg}, L_{Alg+Ch} – buns with lyophilised alginate or alginate-chitosan capsules.
